# Following the Clues: Usefulness of Biomarkers of Neuroinflammation and Neurodegeneration in the Investigation of HTLV-1-Associated Myelopathy Progression

**DOI:** 10.3389/fimmu.2021.737941

**Published:** 2021-10-26

**Authors:** Flávia dos Santos Souza, Nicole Lardini Freitas, Yago Côrtes Pinheiro Gomes, Rafael Carvalho Torres, Juliana Echevarria-Lima, Isaac Lima da Silva-Filho, Ana Claudia Celestino Bezerra Leite, Marco Antonio Sales Dantas de Lima, Marcus Tulius Teixeira da Silva, Abelardo de Queiroz Campos Araújo, Otávio Melo Espíndola

**Affiliations:** ^1^ Laboratório de Pesquisa Clínica em Neuroinfecções, Instituto Nacional de Infectologia Evandro Chagas (INI), Fundação Oswaldo Cruz (FIOCRUZ), Rio de Janeiro, Brazil; ^2^ Seção de Imunodiagnóstico, Instituto Nacional de Infectologia Evandro Chagas (INI), Fundação Oswaldo Cruz (FIOCRUZ), Rio de Janeiro, Brazil; ^3^ Plataforma de Imunoanálises, Instituto de Biofísica Carlos Chagas Filho (IBCCF), Universidade Federal do Rio de Janeiro (UFRJ), Rio de Janeiro, Brazil; ^4^ Serviço de Citometria de Fluxo, Instituto de Puericultura e Pediatria Martagão Gesteira, Universidade Federal do Rio de Janeiro (UFRJ), Rio de Janeiro, Brazil; ^5^ Laboratório de Imunologia Básica e Aplicada, Instituto de Microbiologia Paulo de Góes, Universidade Federal do Rio de Janeiro (UFRJ), Rio de Janeiro, Brazil; ^6^ Serviço de Neurologia, Hospital Universitário Clementino Fraga Filho, Universidade Federal do Rio de Janeiro (UFRJ), Rio de Janeiro, Brazil; ^7^ Instituto de Neurologia Deolindo Couto (INDC), Universidade Federal do Rio de Janeiro (UFRJ), Rio de Janeiro, Brazil

**Keywords:** HTLV-1, biomarkers, neuroinflammation, neopterin, chemokines, Tau protein, neurofilament, cellular prion protein

## Abstract

Human T-lymphotropic virus type 1 (HTLV-1)-associated myelopathy/tropical spastic paraparesis (HAM/TSP) is a neurodegenerative disease due to axonal damage of the corticospinal secondary to an inflammatory response against infected T-cells. In the present work, we aimed to evaluate biomarkers of neurodegeneration and neuroinflammation in the definition of HAM/TSP prognosis. Neurofilament light (NfL) and phosphorylated heavy (pNfH) chains, total Tau protein, cellular prion protein (PrPc), inflammatory chemokines, and neopterin were quantified in paired cerebrospinal fluid (CSF) and serum samples from HAM/TSP patients (n=21), HTLV-1 asymptomatic carriers (AC) (n=13), and HTLV-1 seronegative individuals with non-inflammatory non-degenerative neurological disease (normal-pressure hydrocephalus) (n=9) as a control group. HTLV-1 proviral load in peripheral blood mononuclear cells and the expression of chemokine receptors CCR4, CCR5, and CXCR3 in infected CD4^+^ T-cells (HTLV-1 Tax^+^ cells) were also assessed. CSF levels of Tau, NfL, and pNfH were similar between groups, but PrPc and neopterin were elevated in HAM/TSP patients. Most individuals in the control group and all HTLV-1 AC had CSF/serum neopterin ratio < 1.0, and two-thirds of HAM/TSP patients had ratio values > 1.0, which positively correlated with the speed of disease progression and pNfH levels, indicating active neuroinflammation. HAM/TSP patients showed high serum levels of CXCR3-binding chemokines (CXCL9, CXCL10, and CXCL11) and elevated CSF levels of CCL2, CCL3, CCL4, CCL17, CXCL5, CXCL10, and CXCL11. Indeed, CXCL10 concentration in CSF of HAM/TSP patients was 5.8-fold and 8.7-fold higher in than in HTLV-1 AC and controls, respectively, and correlated with CSF cell counts. HAM/TSP patients with typical/rapid disease progression had CSF/serum CXCL10 ratio > 1.0 and a higher frequency of CXCR3^+^Tax^+^CD4^+^ T-cells in blood, which indicated a positive gradient for the migration of infected cells and infiltration into the central nervous system. In conclusion, the slow progression of HAM/TSP abrogates the usefulness of biomarkers of neuronal injury for the disease prognosis. Thus, markers of inflammation provide stronger evidence for HAM/TSP progression, particularly the CSF/serum neopterin ratio, which may contribute to overcome differences between laboratory assays.

## Introduction

HTLV-1 infection is endemic in Japan, the Caribbean, Middle East, sub-Saharan Africa, Australia, and South America, particularly in Brazil, and it is estimated that 5 to 10 million people are infected worldwide (1). However, only 0.5−5% of infected individuals develop a neurological disease known as HTLV-1-associated myelopathy/tropical spastic paraparesis (HAM/TSP) after 20 to 40 years of infection (2, 3).

HAM/TSP is an inflammatory disorder that affects the spinal cord, causing axonal degeneration and perivascular demyelination that results in a slowly progressive neurological disability and motor impairment without signs of remission (2, 3). HAM/TSP mainly affects the thoracic segment of the spinal cord. Clinical progression is characterized by hyperreflexia, spasticity and muscular weakness of the lower limbs, which increases the risk of falls, in addition to intestinal and bladder disturbance due to the loss of sphincter control, therefore dramatically impacting the quality of life of the patients (2, 4). A fast decline of the neurological condition can be observed in a small proportion of cases, with loss of motor function and restriction to a wheelchair within two years from symptoms onset (5). Moreover, disease progression is faster in women, particularly after the first pregnancy and before menopause (6, 7). Although muscle weakness and spasticity are potential predictors of HAM/TSP progression, a proportion of patients can present with an incomplete pyramidal syndrome, therefore raising the need for laboratory markers to confirm the disease diagnosis (8, 9).

HAM/TSP results from axonal injury caused by neurotoxic effects of inflammatory cytokines, such as interferon γ (IFN-γ) and tumor necrosis factor α (TNF-α), which are released during anti-HTLV-1 responses by CD8^+^ cytotoxic T lymphocytes (CTLs) against infected CD4^+^ T-cells in the central nervous system (CNS) (10–15). At the early stages of the disease, spinal cord lesions have a detectable expression of HTLV-1 *tax* gene, and an infiltrate of CD4^+^ T-cells and macrophages. Conversely, at late stages (over four years), there is a predominance of CD8^+^ CTLs and low virus expression (3, 16). It has been shown that migration of these cells to the CNS is mainly regulated by IFN-γ-induced chemokines, such as the IFN-γ-induced protein 10 (IP-10)/CXCL10 and the monokine induced by IFN-γ (MIG)/CXCL9, which are ligands of the CXC chemokine receptor 3 (CXCR3) (15, 17).

High HTLV-1 proviral load (PVL) in peripheral blood mononuclear cells (PBMCs) has been shown as a risk factor for the development of HAM/TSP (18–20). Indeed, HAM/TSP patients have higher PVL in the cerebrospinal fluid (CSF) than in blood (21). Conversely, absolute HTLV-1 PVL in peripheral blood or CSF appears to present a low predictive value as a biomarker of HAM/TSP progression (22).

Biomarkers of neurodegeneration have been defined for distinct neurological disorders such as multiple sclerosis, Alzheimer’s disease, amyotrophic lateral sclerosis (ALS), and HIV-associated neurocognitive disorders (HAND) (23–27). In this context, the neurofilament light (NfL) and phosphorylated heavy (pNfH) chains represent key biomarkers of neuronal death since they are the main structural components of myelinated axons (27). Elevated CSF levels of NfL indicate axonal damage in progressive primary and secondary multiple sclerosis and a poor prognosis in early remitting-relapsing multiple sclerosis (28, 29). In HIV carriers, NfL concentration in the CSF correlates with neopterin and albumin levels, suggesting an association between neuronal damage, neuroinflammation, and increased permeability of the blood-brain barrier (BBB), indicating even subclinical neurological injury (30). Another structural component of neurons cytoskeleton is Tau protein, which is primarily expressed in the non-myelinated cortex. Tau protein hyperphosphorylation leads to its dissociation from microtubules, causing axonal instability and formation of fibrillary tangles characteristic of tauopathies such as Alzheimer’s disease (31).

The development of many neurodegenerative diseases has also been associated with disturbs in the cellular prion protein (PrPc), including the Creutzfeldt-Jakob disease and other diseases such as schizophrenia, bipolar disorder, and major depression (32–35). PrPc is a glycoprotein anchored to the outer plasma membrane by a glycosylphosphatidylinositol, and upon cleavage, can be detected in the CSF, serum, and lymph (33). PrPc is constitutively expressed by distinct cell types, including immune and CNS cells, and it is involved in apoptosis, signal transduction, oxidative stress, and in establishment of neuronal synapses (33, 36, 37). Increased concentration of soluble PrPc was shown in the CSF of patients with HAND. Moreover, elevated monocyte chemoattractant protein 1 (MCP-1)/CCL2 and interleukin (IL)-6 levels were seen in PrPc-stimulated astrocytes *in vitro* (38). To date, there are no studies addressing the quantification of soluble PrPc in the CSF and serum of HAM/TSP patients, except for one case with unaltered CSF concentration (35).

The speed of HAM/TSP progression is typically defined by clinical follow-up and consecutive assessment of the neurological disability using scales to determine the motor impairment (3). The CSF levels of biomarkers of inflammation such as CXCL10 and neopterin have been shown to strongly correlate with the speed of HAM/TSP progression, offering a promising application for disease prognosis (39–41). Thus, this study aimed to evaluate the usefulness of neuronal injury, inflammation, and cell migration biomarkers to determine the degree of CNS involvement in HTLV-1-infected individuals. For this purpose, HAM/TSP progression was defined in the context of motor impairment in addition to sensory, urinary, and/or intestinal disturbs evaluated with the IPEC-2 scale.

## Materials and Methods

### Patients and Samples

**Figure 1:**
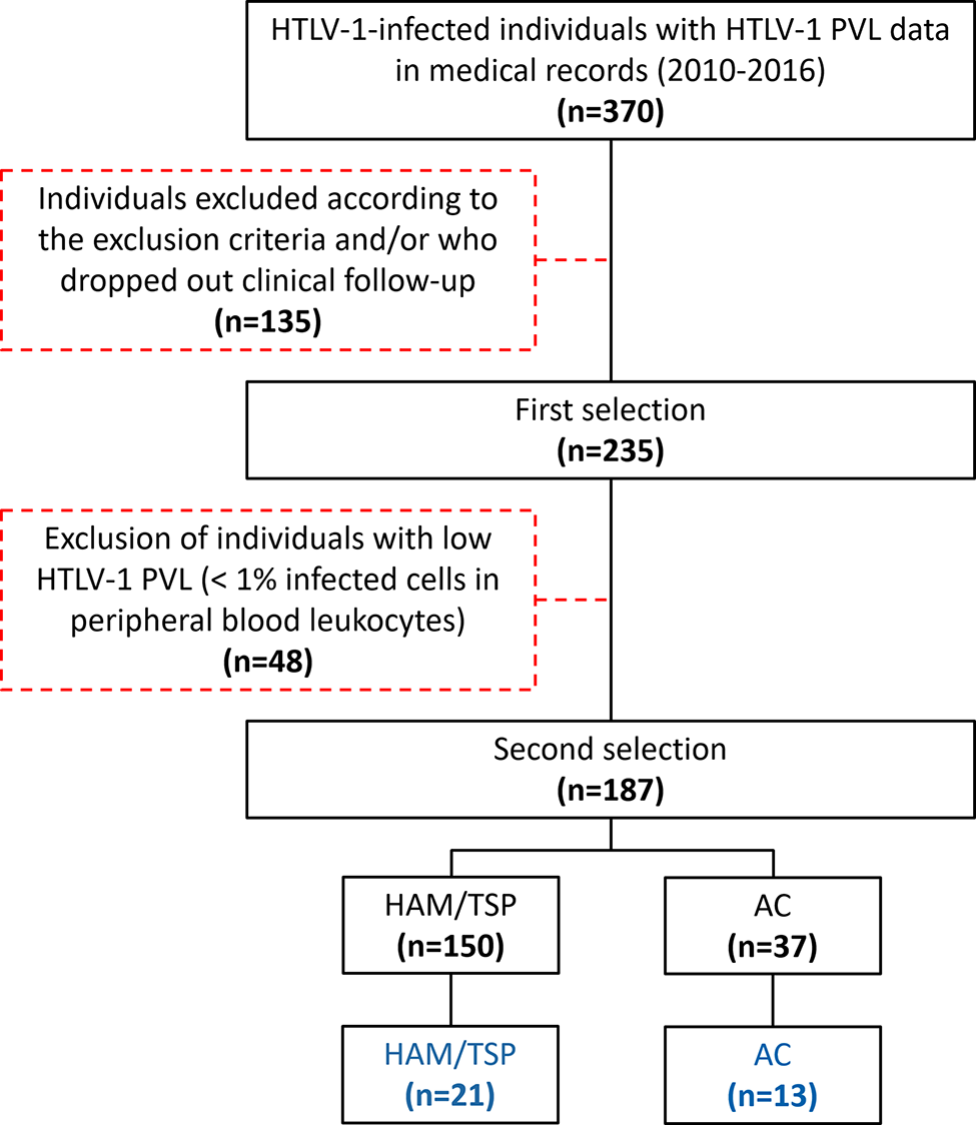
Selection and recruitment of the study population. Patients with HTLV-1 PVL records were accounted and individuals fulfilling the inclusion criteria and on active clinical follow-up were selected. Then, individuals with HTLV-1 PVL < 1% in peripheral blood leukocytes were excluded. Upon recruiting, 21 HAM/TSP patients and 13 AC agreed to participate in the study.

This cross-sectional study was conducted at the Instituto Nacional de Infectologia Evandro Chagas, Rio de Janeiro, Brazil, with the approval of the institutional committee of ethics in research (protocol numbers 53518416.9.0000.5262 and 64426517.8.0000.5262). Patients diagnosed with HAM/TSP according to the World Health Organization’s criteria and HTLV-1 asymptomatic carriers (AC) were selected from an open cohort. Initially, patients with HTLV-1 PVL records and on active clinical follow-up were accounted. Then, individuals with HTLV-1 PVL < 1% in peripheral blood leukocytes were excluded. Upon recruiting, 21 HAM/TSP patients and 13 AC agreed to participate in the study after written informed consent (**Figure 1**). Patients with the following criteria were enrolled in the study: individuals older than 18 years; without a history of autoimmune diseases, lymphoproliferative diseases (ATLL or cutaneous lymphoma), and inflammatory disorders other than HAM/TSP; without diagnostic of other illnesses associated with motor impairment, such as Parkinson’s disease, rheumatoid arthritis, ankylosing spondylitis; not submitted to therapy with corticosteroids (pulse or low oral dose) or other immunomodulatory drugs throughout one year preceding the time at sample collection; no history of pressure ulcers; no concurrent chronic infections such as HTLV-2, HIV, HBV and/or HCV. Paired CSF (n=9) and serum (n=5) samples from HTLV-1 seronegative individuals with non-inflammatory non-degenerative neurological disease (normal-pressure hydrocephalus) were used as non-infected controls.

CSF samples were obtained by lumbar puncture into L3/L4 or L4/L5 vertebrae interspace after anesthesia with 1% lidocaine and were maintained in ice bath. CSF cell counts, glucose, and total protein levels were immediately determined by automated routine analysis. CSF samples were centrifuged at 400 × g for 10 min at 4°C, and the supernatant and cellular sediment were stored at -80°C until use. CSF, serum, and blood in EDTA were collected on the same day within a one-hour interval at the maximum. PBMCs were isolated with Histopaque^®^-1077 (Sigma-Aldrich, United Kingdom) and preserved in liquid nitrogen with 10% dimethyl sulfoxide (Sigma-Aldrich, France) in RPMI-1640 medium (Gibco, United States) with 20% fetal bovine serum (Invitrogen, Brazil).

### Neurological Evaluation

The clinical history was obtained from electronic medical records, and HAM/TSP severity was determined with the IPEC-2 disability scale (**Table 1**).

**Table 1:** IPEC-2 disability scale.

**Motor score: Gait**
0. Normal
1. Abnormal but can walk independently
2. Abnormal and dependent on eventual unilateral support
3. Abnormal and dependent on permanent unilateral support
4. Abnormal and dependent on eventual bilateral support
5. Abnormal and dependent on permanent bilateral support
6. Abnormal, dependent on permanent bilateral support, and occasional use of a wheelchair (WC)
7. Permanent use of a WC, stands up and remains upright without support
8. Permanent use of a WC, uses arms to stand up and remains upright without support
9. Permanent use of a WC, needs assistance from others to stand up and remain upright with support
10. Permanent use of a WC, unable to stand up, exhibits voluntary movements of the lower limbs when seated
11. Permanent use of WC, unable to stand up, and does not have any voluntary movements of the lower limbs
**Motor score: Running**
0. Runs
1. Unable to run
**Motor score: Climbing stairs**
0. Climbs
1. Climbs only when holding the handrail
2. Unable to climb
**Motor score: Jumping**
0. Jumps on one or two feet
1. Jumps on two feet, but not with only one
2. Jumps on two feet only with hand support
3. Unable to jump
**Spasticity score: Clonus**
0. Absent
1. Only induced by the examiner
2. Spontaneous
**Spasticity score: Flexor/extensor spasms**
0. Absent
1. Present
**Sensory score: Paresthesia**
0. Absent
1. Present, eventually
2. Present, permanently
**Sensory score: Lumbar pain**
0. Absent
1. Present, eventually
2. Present during most of the day
**Sensory score: Lower limb pain**
0. Absent
1. Present, eventually
2. Present during most of the day
**Sphincter score: Bladder control**
0. Total
1. Urgency
2. Eventual incontinence or retention
3. Use of permanent catheter or regular use of relieving catheter
**Sphincter score: Bowel continence**
0. Normal
1. Constipation
2. Incontinence or total retention, needs manual extraction or enemas
**Total:** 0–31

The IPEC-2 scale is a variation of IPEC-1 disability scale, which was previously validated (42), and differentiated only by separated assessment of lumbar pain and lower limb pain. IPEC-2 scale assesses motor impairment in addition to sensory, urinary, and intestinal alterations in HAM/TSP. Neurological evaluation was undertaken in a private and quiet room and disability scores were determined by one of four trained and qualified neurologists involved in the study (ACCBL, MASDL, MTTS, AQCA). Before the scale was used, patients were instructed about it and all doubts about the scale were resolved. Clonus was assessed by direct examination, performed by the trained neurologist, always testing both feet and patellae, as done in the classical neurological examination. Flexor and extensor spasms were assessed by direct questioning and also by observation during clinical evaluation. Paresthesia, as well as pain, as subjective symptoms, were assessed by direct questioning. Neurological impairment in HAM/TSP evaluated with the IPEC-2 scale was defined as mild for patients with scores between 1 and 10 points, moderate for scores between 11 and 21 points and severe for scores ≥ 22 points.

Cognitive function was evaluated in all patients as part of the routine neurological examination employing the Mini-Mental State Examination (43); none of the patients included in the study had scores of less than 24 points, which would impair the understanding of the issues involved in the scale.

The speed of disease progression was defined by the quotient between the disability score and the duration of disease (the interval between disease onset and sample collection). The first quartile (≤ 0.37 points/year) represented patients with very slow progression, the second and third quartiles (from 0.38 to 1.44 points/year) consisted of patients with typical progression, and the fourth quartile (≥ 1.45 points/year) presented individuals with rapid progression (44).

### Quantification of HTLV-1 PVL

DNA was extracted from PBMCs and CSF cells with the QIAamp DNA blood mini kit (Qiagen, Germany), following the manufacturer’s instructions, and HTLV-1 PVL was determined as previously described (45). Briefly, quantitative PCR was carried out with the Rotor-Gene Probe PCR kit (Qiagen, Germany) in a Rotor-Gene Q 5-plex instrument (Qiagen, Germany). Independent reactions containing primers and 5’-FAM and 3’-TAMRA-labeled TaqMan^®^ probes (Sigma-Aldrich, United States) for the amplification of the HTLV-1 *tax* and human *β-globin* genes (20) were carried out with 100 ng of DNA, in a final volume of 25 μL. HTLV-1 PVL was calculated as *tax* copies/(*β‐globin* copies/2). The results are shown as the percentage of infected cells in PBMCs and CSF cells.

### Quantification of PrPc and Biomarkers of Neuronal Injury

Commercially available ELISA kits were used to determine CSF levels of soluble PrPc (Human PRNP, Cat #EH2469, FineTest, Wuhan, China), NfL (Human NEFL ELISA, Cat #EH1205, FineTest, Wuhan, China), pNfH (pNF-H ELISA, Cat #RD191138300R, Biovendor, Czech Republic), and total Tau protein (tTau) (Total Tau ELISA, Cat #KHB-0041, Invitrogen, Austria). All assays were carried out following the manufacturer’s instructions. Analyte concentrations were determined in Four-Parameter Logistic Curves. Age-adjusted reference values for NfL (30) and tTau protein (46) in the CSF were used to determine altered levels.

### Quantification of Neopterin and Inflammatory Chemokines

Neopterin concentration in CSF and serum was determined by ELISA (Neopterin ELISA, Cat #RE59325, IBL International, Germany), and the following pro-inflammatory chemokines were quantified with a multiplex cytometry bead-based immunoassay (LEGENDplex™ human pro-inflammatory chemokine panel 13-plex, Biolegend, United States), according to the manufacturer’s instructions: MCP-1/CCL2; Macrophage inflammatory protein 1α (MIP-1α)/CCL3; MIP-1β/CCL4; Regulated upon activation, normal T cell expressed and presumably secreted (RANTES)/CCL5; Eotaxin/CCL11; Thymus and activation-regulated chemokine (TARC)/CCL17; MIP-3α/CCL20; Growth-regulated oncogene α (GRO-α)/CXCL1; Epithelial-derived neutrophil-activating peptide 78 (ENA-78)/CXCL5; IL-8/CXCL8; MIG/CXCL9; IP-10/CXCL10; and Interferon-inducible T-cell alpha chemoattractant (I-TAC)/CXCL11. Data were acquired in a FACSCanto II flow cytometer (BD Biosciences, United States). Reference values for neopterin in serum (< 10 nmol/L) and CSF (< 4.2 nmol/L) (47) were used to define individuals with elevated levels.

### Analysis of HTLV-1 Tax Protein and Chemokine Receptors Expression in CD4+ T-Cells

PBMCs were thawed and washed three times by centrifugation at 300 × g for 10 min at 4°C with RPMI-1640 medium and suspended in phosphate-buffered saline (PBS) with 0.25% bovine serum albumin (BSA) and 2 mM EDTA and kept in ice bath. CD8^+^ T-cell depletion was performed with Dynabeads CD8 (Invitrogen, Norway) according to the manufacturers’ instructions. CD8-depleted PBMCs were suspended in RPMI-1640 medium with 10% fetal bovine serum and plated at 5×10^6^ cells/well in 24-well microplates. Cells were incubated for 20 h at 37°C with 5% CO_2_ atmosphere and then stained with Live/Dead Yellow (Invitrogen, United States) to determine cell viability, following the manufacturers’ instructions. After, cell surface was stained with anti-CCR4-PE-Cy7, anti-CCR5-PE, and anti-CXCR3-APC-Cy7 monoclonal antibodies (mAbs) (all from Biolegend, United States) for 30 min at 4°C. Further, intracellular staining was carried out using the FoxP3 intracellular staining kit (eBioscience, United States), and cells were incubated with anti-CD3-BV785, anti-CD4-efluor450 mAbs (Biolegend, United States), and biotin-conjugated anti-HTLV-1 Tax protein mAb (Clone Lt-4 kindly donated by Dr. Yuetsu Tanaka, Kitasato University, Kanagawa, Japan). After washing, APC-conjugated streptavidin diluted in 1× permeabilization buffer (1:200) was added to tubes, and cells were incubated for 10 min at 4°C. Cells were washed twice with permeabilization buffer, fixed with 1% paraformaldehyde, and analyzed in a CytoFlex S flow cytometer (Beckman-Coulter, United States), with the acquisition of 500,000 events, and data analysis was carried out with CytExpert version 1.2 software (Beckman-Coulter, USA). Briefly, live cells (Live/Dead Yellow unstained cells) were gated, and doublets were excluded according to FSC-A and FSC-H distribution. Lymphocytes were gated in FSC-A and SSC-A plots, and the CD3^+^CD4^+^ T-cells subset was subsequently defined. HTLV-1 Tax expression was evaluated in the population of CD4^+^ T-cells, and the frequency of cells expressing CCR4, CCR5, and CXCR3 chemokine receptors was determined in the subsets of Tax^+^ and Tax^−^ CD4^+^ T-cells.

### Statistical Analysis

Data were analyzed with GraphPad Prism v.5 and R software v.3.6.1. Descriptive analysis included the frequency of categorical variables and summary measures of quantitative variables: mean, median, standard deviation and interquartile range (IQR). Normal distribution of quantitative data was evaluated ​​with the Kolmogorov-Smirnov test. Comparison of parametric data between two groups was performed by Student’s *t*-test, with Welch’s correction for distinct variances when applicable, and between three groups using ANOVA test with Bonferroni correction for multiple comparisons. Analysis of nonparametric data of two groups was carried out by Mann-Whitney test, and by Kruskal-Wallis test with Dunn’s post-test for multiple comparisons when comparing three groups. Correlation analysis was performed by Spearman’s rank correlation. The association between qualitative data was evaluated by Chi-square method. Differences with *p*-value < 0.05 were considered significant. Relative expression of distinct biomarkers between individuals was evaluated by heatmap analysis using the gplots package for *R* software after log_2_ transformation of values. Complete agglomeration method and Euclidean algorithm were used to perform clustering and to obtain the distance matrix, respectively.

## Results

### Characterization of the Study Population

The study population was constituted by 60.5% of women and 39.5% of men, with no difference in the distribution between groups (**Table 2**). The mean age of HTLV-1 AC (62.2 ± 10.2 years), HAM/TSP patients (55.4 ± 13.4 years) and HTLV-1-seronegative controls (60.33 ± 19.74 years) was similar, and HAM/TSP patients were characterized by chronic disease, with a mean of 12.9 ± 8.1 years of disease, varying from 5 months to 29 years (**Table 2**). The CSF of all enrolled individuals had normal glucose levels, 100% mononuclear cells, and no clinically relevant alteration in total protein, although HTLV-1 AC presented higher total protein levels than the control group (**Table 2**, ANOVA *p* < 0.032). All HTLV-1 AC and individuals in the control group presented normal CSF cell counts ​​(≤ 5 cells/mm^3^), with a median of 1 cell/mm^3^, while HAM/TSP patients had higher cell counts, with a median of 4 cells/mm^3^ (**Table 2**, Kruskal-Wallis *p* < 0.001), and 38.1% (n=8) presented pleocytosis. In addition, HAM/TSP patients had higher HTLV-1 PVL than HTLV-1 AC in PBMCs (Student’s *t*-test, *p* = 0.049) and in CSF cells (Student’s *t*-test, *p* = 0.020) (**Table 2**).

**Table 2:** Characterization of the study population.

	Groups			
	HTLV-1 AC	HAM/TSP	Control	*p*-value^i^
	(n=13)	(n=21)	(n=9)	
**Age (years)^a^**	62.2 ± 10.2	55.4 ± 13.4	60.33 ± 19.74	0.367
**Sex at birth^b^**				
Men	6 (14.0%)	8 (18.6%)	3 (7.0%)	0.818
Women	7 (16.3%)	13 (30.2%)	6 (14.0%)	
**Disease duration (years)^a,c^**	n.a.	12.9 ± 8.1	n.a.	n.a.
**IPEC-2 disability scale**				
mild (1-10 points)	n.a.	13 (61.9%)	n.a.	n.a.
moderate (11-21 points)	n.a.	5 (23.8%)	n.a.	n.a.
severe (≥22 points)	n.a.	3 (14.3%)	n.a.	n.a.
**Sensory and sphincter disturbs**				
Paresthesia	n.a.	7 (33.3%)	n.a.	n.a.
Lumbar pain	n.a.	9 (42.9%)	n.a.	n.a.
Lower limb pain	n.a.	11 (52.4%)	n.a.	n.a.
Urinary disturbances	n.a.	15 (71.4%)	n.a.	n.a.
Bowel continence	n.a.	15 (71.4%)	n.a.	n.a.
**HTLV-1 PVL in PBMCs (%)^d^**	4.50 ± 3.64	8.46 ± 6.37	n.a.	**0.049**
**CSF^e^**				
Glucose (mg/dL)^a^	63.00 ± 11.52	61.67 ± 8.97	54.78 ± 8.64	0.133
Total protein (mg/dL)^a^	48.25 ± 13.06	43.99 ± 12.46	34.22 ± 8.06	**0.032**
Cell counts (cells/mm^3^)^f,g^	1 (1 – 2)	4 (1.5 - 7.5)	1 (1 – 1)	**<0.001**
HTLV-1 PVL (%)^d,h^	9.16 ± 0.82	18.13 ± 9.99	n.a.	**0.020**

AC, asymptomatic carriers; HAM/TSP, HTLV-1-associated myelopathy/tropical spastic paraparesis; n.a., not applicable; PVL, proviral load; PBMCs, peripheral blood mononuclear cells; CSF, cerebrospinal fluid; IQR, interquartile range 25%-75%. ^a^ Mean ± standard deviation. Statistical analysis performed with ANOVA test with Bonferroni correction for multiple comparisons. ^b^ Statistical analysis performed with Chi-square test. ^c^ Interval between the onset of pyramidal syndrome and sample collection. ^d^ Mean ± standard deviation. Statistical analysis was performed with Student’s t-test ^e^ Reference values for CSF: glucose, 50-80 mg/dL; total protein, 15-45 mg/dL; cell counts, < 5 cells/mm^3^. ^f^ Median values and IQR. Statistical analysis performed with Kruskal-Wallis test with Dunn’s post-test for multiple comparisons. ^g^ All samples had 100% mononuclear cell infiltrate. ^h^ HTLV-1 PVL was quantified in CSF cells of HTLV-1 AC (n = 3) and HAM/TSP patients (n = 10). ^i^ p-value < 0.05 was significant (in bold).

**Figure 2:**
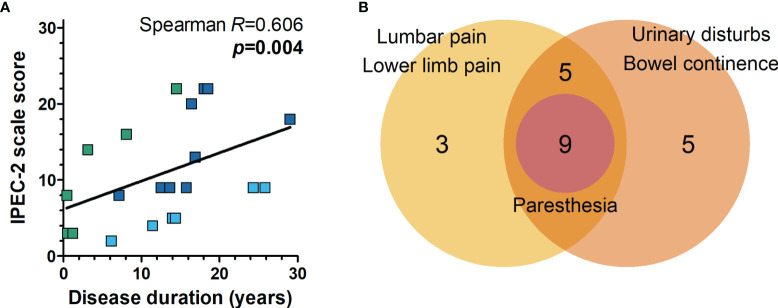
Neurological impairment associated with HAM/TSP. **(A)** The severity of neurological disability was evaluated with the IPEC-2 scale, and the correlation with disease duration (the interval between disease onset and sample collection) was performed with Spearman’s correlation rank. HAM/TSP patients were identified according to the speed of disease progression as very slow (light blue), typical/slow (dark blue) and rapid (green). **(B)** Combined frequency of patients with multiple neurological manifestations other than motor impairment in HAM/TSP. All patients with paresthesia presented with lumbar and/or lower limb pain in addition to urinary and/or bowel disturbances.

Motor impairment and other neurological manifestations, such as sensory alterations, muscle tone, urinary disturbance, and fecal continence in HAM/TSP, were assessed with the IPEC-2 scale. Most HAM/TSP patients included in the study had mild impairment (61.9%, n=13), while 23.8% (n=5) had moderate, and 14.3% (n=3) had severe neurological involvement (**Table 2**). Urinary and/or bowel disturbances were present in 71.4% of HAM/TSP patients. Sensory alterations such as paresthesia, lumbar pain, and lower limb pain were seen in 33.3%, 42.9%, and 52.4% of patients, respectively (**Table 2**). The severity of neurological impairment was related to the disease duration (**Figure 2A**, Spearman *R* = 0.606, *p* = 0.004). Additionally, at least one of extra motor manifestations was reported in all HAM/TSP patients (**Figure 2B**).

### Biomarkers of Neurodegeneration Fail to Distinct HTLV-1 AC and HAM/TSP Patients

**Figure 3:**
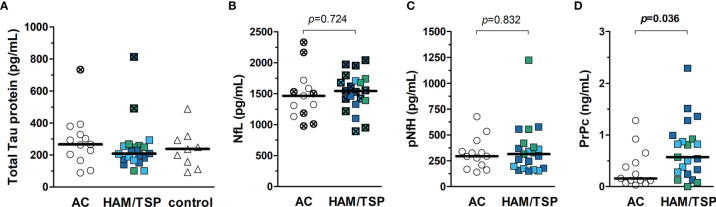
Biomarkers of neurodegeneration in the CSF of HTLV-1-infected individuals. **(A)** Total Tau protein, **(B)** neurofilament light chain (NfL), **(C)** phosphorylated neurofilament heavy chain, and **(D)** cellular prion protein (PrPc) were quantified by ELISA in the CSF of HTLV-1 asymptomatic carriers (AC) (n = 13) and HAM/TSP patients (n = 21). **(A)** A control group of HTLV-1 seronegative individuals (n = 9) was included, and the comparison between groups was performed by Kruskal-Wallis test with Dunn’s post-test for multiple comparisons. **(B–D)** Statistical analysis was performed with Mann-Whitney test. A *p*-value < 0.05 was considered significant. HAM/TSP patients were identified according to the speed of disease progression as very slow (light blue), typical/slow (dark blue) and rapid (green). Individuals with altered levels of total Tau protein and NfL according to age-corrected reference values are shown with bold symbols marked with an ×. Reference values for NfL in CSF defined by Jessen Krut et al. (30): 18-30 years, < 380 pg/mL; 30-39 years, < 560 pg/mL; 40-59 years, < 890 pg/mL; > 59 years-old, < 1850 pg/mL. Reference values for total Tau protein determined by Sjögren et al. (46): 21-50 years, < 300 pg/mL; 51-70 years, < 450 pg/mL; 71-93 years, < 500 pg/mL.

The CSF of HTLV-1 AC and HAM/TSP patients was examined to determine whether biomarkers of neuronal injury might predict the neurological involvement in HAM/TSP. However, no difference was seen between groups considering the concentration of tTau, NfL, and pNfH (**Figures 3A** **–C**, respectively). Conversely, PrPc levels were increased in the CSF of HAM/TSP patients (**Figure 3D**, Mann-Whitney *p* = 0.036). Individuals with altered CSF levels of tTau and NfL according to age were observed in both groups. While one HTLV-1 AC and two HAM/TSP patients had altered tTau, elevated NfL concentration was observed in 53.8% (7 of 13) of HTLV-1 AC and 66.7% (14 of 21) of HAM/TSP patients.

### Neopterin as a Biomarker of Active CNS Inflammation in HTLV-1 Infected Individuals

**Figure 4:**
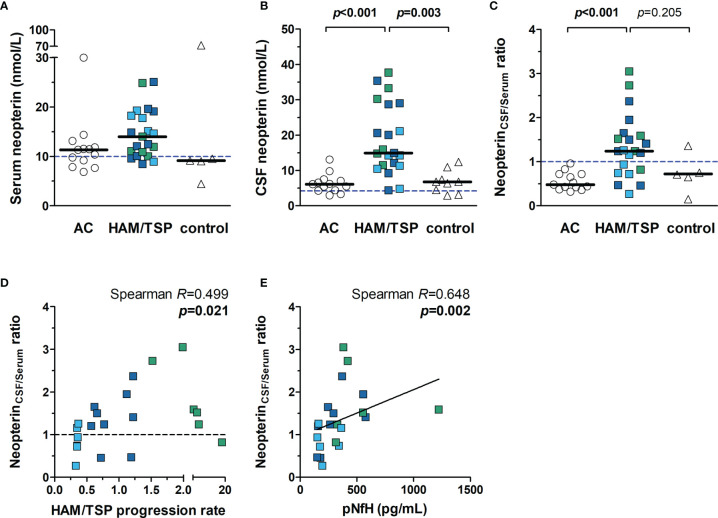
Neopterin concentration in the serum and CSF of HTLV-1-infected individuals. Neopterin was quantified by ELISA in the **(A)** serum and **(B)** CSF of HTLV-1 asymptomatic carriers (AC) (n=13) and HAM/TSP patients (n=21). HAM/TSP patients were identified according to the speed of disease progression as very slow (light blue), typical/slow (dark blue) and rapid (green). **(C)** The ratio between CSF and serum neopterin concentration was calculated for each individual, and those with values > 1.0 were considered with active central nervous system inflammation. Serum (n=5) and CSF (n=9) samples of HTLV-1 seronegative individuals were included as controls. Statistical analysis was performed with Kruskal-Wallis test with Dunn’s post-test for multiple comparisons, and *p*-value < 0.05 was considered significant. **(D)** Spearman’s correlation rank was used to evaluate the association between neopterin_CSF/Serum_ ratio and HAM/TSP progression rate (points/year), defined as the quotient between the score in IPEC-2 disability scale and the time of disease in years. **(E)** Correlation between neopterin_CSF/Serum_ ratio and CSF levels of phosphorylated neurofilament heavy chain (pNfH), a biomarker of axonal degeneration. Dashed lines indicate reference values for **(A)** serum neopterin (< 10 nmol/L), **(B)** CSF neopterin (< 4.2 nmol/L), and **(C, D)** neopterin_CSF/Serum_ ratio (< 1.0).

The median concentration of neopterin in the serum was similar between HAM/TSP patients (14.01 nmol/L, IQR 10.48 - 18.67 nmol/L), HTLV-1 AC (11.34 nmol/L, IQR 8.53 - 12.48 nmol/L) and HTLV-1-seronegative individuals (9.16 nmol/L, IQR 6.74 - 40.74 nmol/L) (**Figure 4A**, Kruskal-Wallis *p* = 0.059). Elevated neopterin in serum was observed in 85.7% (18 in 21) of HAM/TSP patients. However, 61.5% (8 in 13) of HTLV-1 AC also had elevated levels, and only one individual had an altered concentration in the control group. Neopterin levels were significantly higher in the CSF of HAM/TSP patients (14.93 nmol/L, IQR 11.40 - 28.85 nmol/L) compared to HTLV-1 AC (6.11 nmol/L, IQR 4.32 - 7.26) and HTLV-1-seronegative individuals (6.80 nmol/L, IQR 3.81 - 9.16 nmol/L) (**Figure 4B**). All HAM/TSP patients presented elevated neopterin in CSF. However, 84.6% (11 in 13) of HTLV-1 AC and 77.8% (7 in 9) HTLV-1-seronegative individuals also had increased levels.

The speed of disease progression has been shown to correlate with increasing neopterin levels in CSF, and upper and lower cut-off values have been proposed (40). HAM/TSP patients with neopterin ≥ 44 nmol/L are possible rapid progressors, and those with neopterin < 5.5 nmol/L would likely display very slow disease progression. Consequently, patients within these upper and lower cut-offs would be characterized by the typically slow course of HAM/TSP. None of the patients in the study had CSF neopterin ≥ 44 nmol/L. Only two patients showed values below 5.5 nmol/L, but one of them had typical HAM/TSP progression.

Neopterin is a byproduct of inflammatory responses associated with monocytes/macrophages and dendritic cells. The BBB is selective for neopterin, and therefore the CSF concentration of neopterin is unrelated to blood levels (47, 48). Thus, a ratio between the CSF and serum neopterin levels (neopterin_CSF/serum_) was calculated to define the degree of CNS inflammation. All HTLV-1 AC had values < 1.0, indicating that inflammation was more pronounced or restricted to the periphery, with a median of 0.48 (IQR 0.42 - 0.72), and HTLV-1-seronegative individuals presented a median of 0.71 (IQR 0.40 - 1.06) (**Figure 4C**). On the other hand, HAM/TSP patients had a median neopterin_CSF/serum_ ratio of 1.24 (IQR 0.82 - 1.59), which was significantly higher (**Figure 4C**), showing that active CNS inflammation was present in two-thirds (14 of 21) of HAM/TSP patients.

HAM/TSP progression correlated with neopterin_CSF/serum_ ratio (**Figure 4D**, Spearman *R* = 0.499, *p* = 0.021), indicating that the speed of disease progression is related to the intensity of CNS inflammation. Although neopterin_CSF/serum_ ratio differentiated most individuals according to the speed of disease progression, it better indicated the disease activity, which is supported by the positive correlation with CSF levels of pNfH (**Figure 4E**, Spearman *R* = 0.648, *p* = 0.002).

### Inflammatory Chemokines in the CSF and Serum of HTLV-1-Infected Individuals

**Figure 5:**
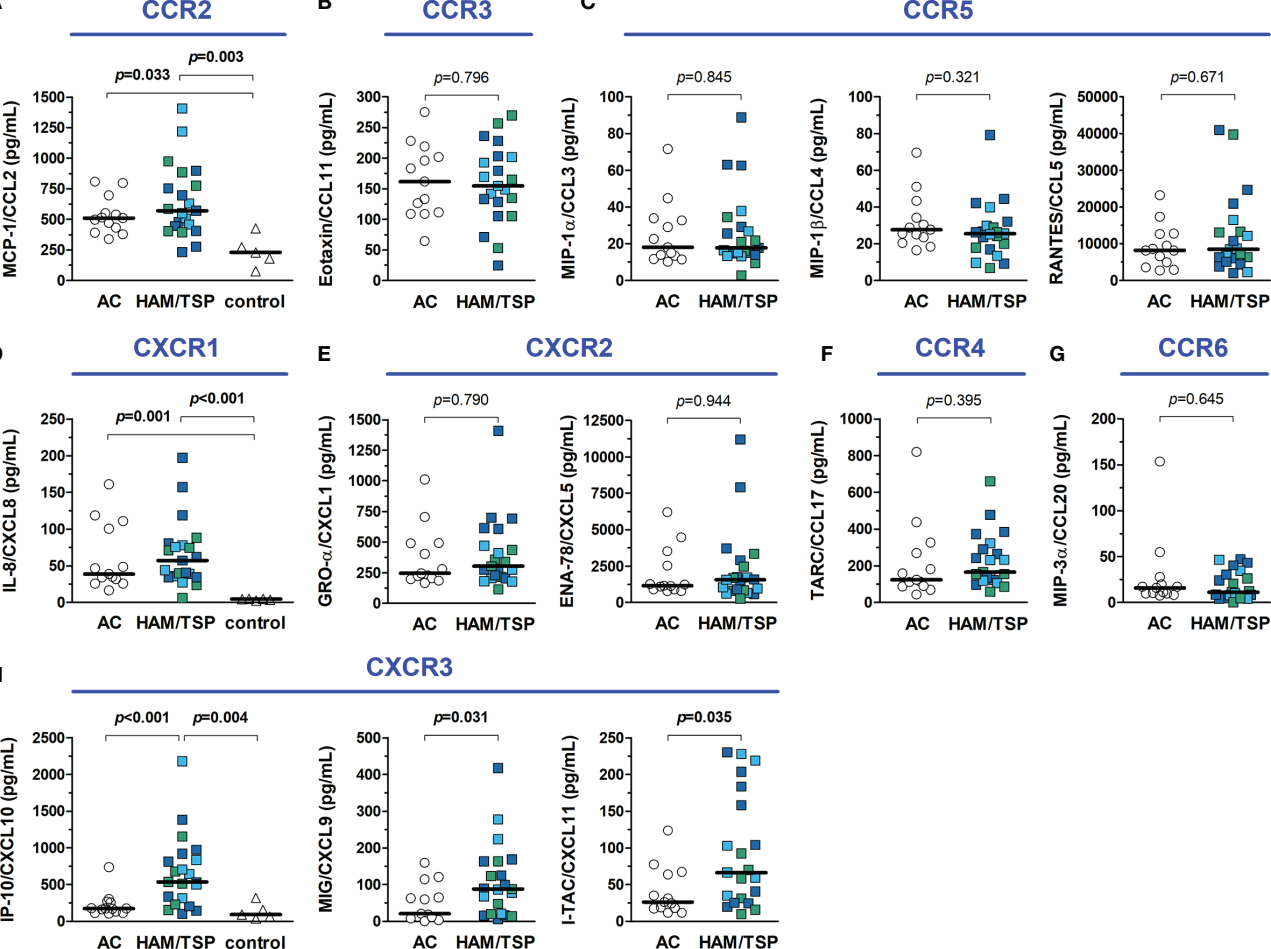
Inflammatory chemokines in the serum of HTLV-1 asymptomatic carriers (AC) and HAM/TSP patients. Chemokines were quantified with a multiplex cytometry bead-based immunoassay, and graphs were organized according to their respective receptors, shown in blue. **(A)** CCL2 **(B)**, CCL11, **(C)** CCL3, CCL4 and CCL5, **(D)** CXCL8, **(E)** CXCL1 and CXCL5, **(F)** CCL17, **(G)** CCL20, and **(H)** CXCL9, CXCL10 and CXCL11. HAM/TSP patients were identified according to the speed of disease progression as very slow (light blue), typical/slow (dark blue) and rapid (green). Comparisons between two groups were performed with Mann-Whitney test, and the analysis between three groups was carried out with Kruskal-Wallis test with Dunn’s post-test for multiple comparisons. Differences with p-value < 0.05 were considered significant.

Chemokines are proteins with cell-type specific chemotactic functions and are involved in cell homing and recruitment to sites of inflammation. It was observed that serum levels of CCL2, CCL3, CCL4, CCL5, CCL11, CCL17, CCL20, CXCL1, CXCL5, and CXCL8 had no significant differences between groups (**Figure 5**). Conversely, serum levels of CXCL9, CXCL10, and CXCL11, which are ligands of the chemokine receptor CXCR3, were increased in HAM/TSP patients (**Figure 5**). In addition, serum CCL2 and CXCL8 levels were higher in both HTLV-1 AC and HAM/TSP patients compared with HTLV-1-seronegative controls (**Figure 5**).

**Figure 6:**
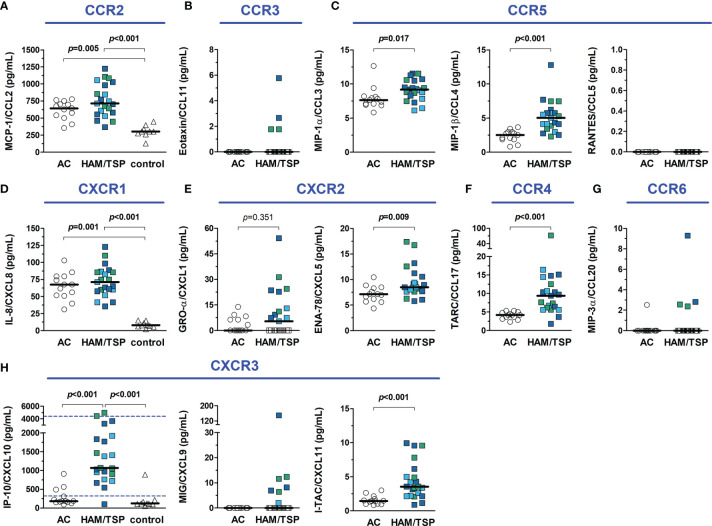
Inflammatory chemokines in the cerebrospinal fluid (CSF) of HTLV-1 asymptomatic carriers (AC) and HAM/TSP patients. Chemokines were quantified with a multiplex cytometry bead-based immunoassay, and graphs were organized according to their respective receptors, shown in blue. **(A)** CCL2 **(B)**, CCL11, **(C)** CCL3, CCL4 and CCL5, **(D)** CXCL8, **(E)** CXCL1 and CXCL5, **(F)** CCL17, **(G)** CCL20, and **(H)** CXCL9, CXCL10 and CXCL11. HAM/TSP patients were identified according to the speed of disease progression as very slow (light blue), typical/slow (dark blue) and rapid (green). Comparisons between two groups were performed with Mann-Whitney test, and the analysis between three groups was carried out with Kruskal-Wallis test with Dunn’s post-test for multiple comparisons, and differences with p-value < 0.05 were considered significant. Dashed lines in the graph for IP-10/CXCL10 data represent the upper and lower cut-offs for CXCL10 concentration in the CSF to predict the speed of HAM/TSP progression as very slow (< 320 pg/mL), typical/slow (320–4,399 pg/mL), and rapid (≥ 4,400 pg/mL), as proposed by Sato et al. (40).

The CSF concentration of distinct inflammatory chemokines is shown in **Figure 6**. CCL5 was undetectable (< 3.41 pg/mL) in all individuals. Moreover, all HTLV-1 AC had undetectable levels of CCL11 (< 1.68 pg/mL) and CXCL9 (< 1.36 pg/mL). Indeed, low levels of CCL11 and CCL20 were present in 4 of 21 (19%) HAM/TSP patients, and CXCL9 was detected in the CSF of only 9 of 21 (43%) HAM/TSP patients. No difference between groups was observed for CXCL1. However, CCL2, CCL3, CCL4, CCL17, CXCL5, CXCL10, and CXCL11 were significantly elevated in HAM/TSP patients (**Figure 6**). CSF levels of CXCL10 in HAM/TSP patients (1,067.5 pg/mL, IQR 830.5 - 1,876.0 pg/mL) were 5.8 and 8.7-fold higher than in HTLV-1 AC (182.6 pg/mL, IQR 155.2 - 401.9 pg/mL) and HTLV-1-seronegative controls (122.3 pg/mL, IQR 105.6 - 189.2 pg/mL), respectively (**Figure 6**). Moreover, CSF concentration of CCL2 and CXCL8 were significantly higher in both groups of HTLV-1 infected individuals (AC and HAM/TSP patients) in comparison with controls (**Figure 6**).

The correlation analysis between the concentration of chemokines showed similar patterns in the serum of HTLV-1 AC and HAM/TSP patients, likely associated with immune responses against the infection (**Supplementary Figure 1**). However, the CSF of HAM/TSP patients showed widespread correlation, which in turn was virtually absent in HTLV-1 AC (**Supplementary Figure 2**). This corroborates the observation of many chemokines with elevated levels in the CSF of HAM/TSP patients, suggesting that CNS inflammation in these individuals triggers several and possibly redundant mechanisms of immune response.

It has been shown that CSF levels of CXCL10 might predict the speed of HAM/TSP progression. Thus, upper (4,400 pg/mL) and lower (320 pg/mL) cut-offs were proposed (40). Considering this, two patients in the study would be classified as rapid progressors, one with very slow HAM/TSP progression and most patients with typical HAM/TSP. Although both patients classified as rapid progressors had fast neurological deterioration as evaluated with the IPEC-2 scale, the patient identified with very slow disease progression had, in fact, a typical HAM/TSP course (**Figure 6**).

**Figure 7:**
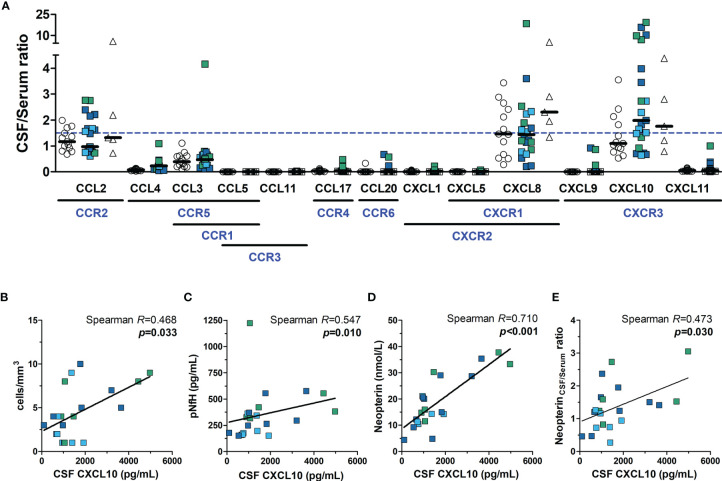
Neuroinflammatory activity in HTLV-1 asymptomatic carriers (AC) and HAM/TSP patients. **(A)** The ratio between the CSF and serum concentration of chemokines was calculated, and values ≥ 1.5 (dashed blue line) indicated the direction of a positive gradient to control the migration of immune cells to the central nervous system. Chemokine receptors are shown in blue under their respective ligands. HAM/TSP patients were identified according to the speed of disease progression as very slow (light blue), typical/slow (dark blue) and rapid (green). Spearman’s correlation rank was used to evaluate the association between CSF levels of CXCL10 and **(B)** CSF cell counts, **(C)** phosphorylated neurofilament heavy chain (pNfH), **(D)** CSF levels of neopterin, and **(E)** the neopterin_CSF/Serum_ ratio, and *p*-value < 0.05 was considered significant.

The CSF to serum ratio concentration of chemokines was calculated, and values ≥ 1.5 (CSF concentration 50% higher than in serum) were considered suggestive of a positive gradient to attract immune cells to the CNS. A proportion of HTLV-1 AC and HAM/TSP patients presented CCL2, CXCL8, and/or CXCL10 levels in the CSF higher than in serum, which was also observed among HTLV-1-seronegative controls (**Figure 7A**). However, increasing CXCL10 levels in the CSF of HAM/TSP patients were associated with elevated CSF cells counts (**Figure 7B**, Spearman *R* = 0.468, *p* = 0.033), higher pNfH levels (**Figure 7C**, Spearman *R* = 0.547, *p* = 0.010), elevated neopterin in the CSF (**Figure 7D**, Spearman *R* = 0.710, *p* < 0.001), and increased neopterin_CSF/serum_ ratio (**Figure 7E**, Spearman *R* = 0.473, *p* = 0.030). A correlation was also seen between the CXCL10_CSF/serum_ ratio and the disease progression rate (Spearman *R* = 0.476, *p* = 0.034), although it did not differentiate between individuals with very slow and typical or rapid progression (data not shown). Moreover, higher CSF levels of neopterin in HAM/TSP patients were associated with elevated CSF concentration of CCL2 (Spearman *R* = 0.466, *p* = 0.033), and CXCL8 (Spearman *R* = 0.719, *p* < 0.001).

### HAM/TSP Patients Show a Higher Frequency of CXCR3+CD4+ T-Cells in Peripheral Blood

**Figure 8:**
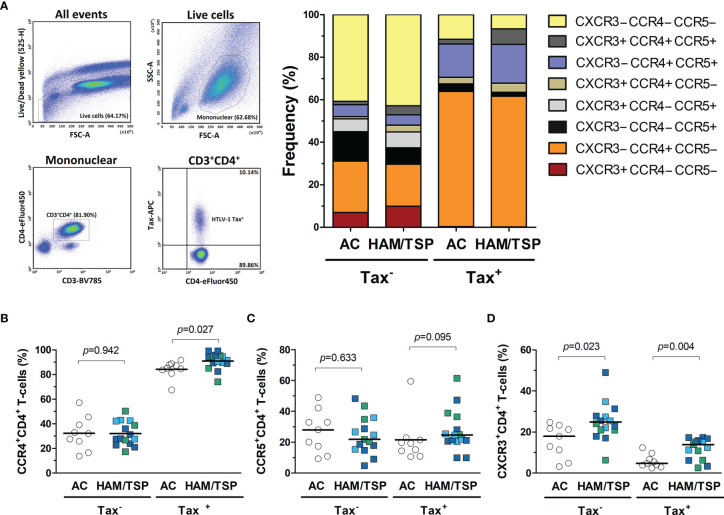
Expression of chemokine receptors by CD4^+^ T-cells from HTLV-1-infected individuals. **(A)** Infected (Tax^+^) and uninfected (Tax^−^) CD4^+^ T-cells from the peripheral blood of HTLV-1 asymptomatic carriers (AC) (n = 9) and HAM/TSP patients (n = 15) were identified by flow cytometry analysis after intracellular staining of HTLV-1 Tax protein in CD8^+^ T-cell-depleted PMBCs cultured for 20 h without stimulation. Live cells were gated, and CD3^+^CD4^+^ T-cells were selected within the subset of mononuclear cells. The expression profile of the chemokine receptors CCR4, CCR5, and CXCR3 was evaluated within the subsets of Tax^+^ and Tax^−^ CD4^+^ T-cells. The frequency of cells expressing **(B)** CCR4, **(C)** CCR5, and **(D)** CXCR3 was determined within the populations of Tax^+^ and Tax^−^ CD4^+^ T-cells, and the comparison between AC and HAM/TSP patients was performed with Student’s *t*-test. Differences with *p*-value < 0.05 were considered significant. HAM/TSP patients were identified according to the speed of disease progression as very slow (light blue), typical/slow (dark blue) and rapid (green).

HAM/TSP development is associated with a strong Th1 response in the CNS (3, 17). Therefore, the expression of Th1-related CXCR3 and CCR5 chemokine receptors was evaluated in CD4^+^ T-cells from peripheral blood. In addition, the expression of CCR4, a chemokine receptor induced in HTLV-1-infected cells, was also assessed (49, 50). HTLV-1 Tax protein expression was used to identify infected cells and determine whether HAM/TSP patients have a greater frequency of infected CD4^+^ T-cells with the potential to infiltrate the CNS. As expected, the frequency of Tax^+^ cells in CD4^+^ T-cells positively correlated with the HTLV-1 PVL (Spearman *R* = 0.662, *p* < 0.001). According to Tax expression, distinct patterns of CCR4, CCR5, and CXCR3 expression in CD4^+^ T cells were observed (**Figure 8A**). Most Tax^+^ CD4^+^ T-cells were CCR4^+^ in both patient groups (**Figure 8B**). No difference was observed in the expression of CCR4 and CCR5 in Tax−CD4^+^ T-cells (**Figures 8B** and 8C, respectively), as well as in CCR5 expression by Tax^+^ CD4^+^ T-cells (**Figure 8C**). In contrast, HAM/TSP patients had a higher frequency of CXCR3^+^ cells in the subset of Tax − and Tax^+^ CD4^+^ T-cells (**Figure 8D**). No correlation was observed between the frequency of CCR4, CCR5 and CXCR3 expression in Tax^+^ and Tax − CD4^+^ T-cells and the concentration of their respective ligands in the CSF and serum (**Supplementary Figure 3**). However, these data corroborate with the role of CXCR3 in directing the migration of both infected and inflammatory CD4^+^ T-cells into the CNS, particularly through CXCL10 signaling, which is at markedly high levels in the CNS of HAM/TSP patients. However, cellular migration mediated by CCR5 in response to CCL3/CCL4 may also play a role in HAM/TSP.

### Distinct Profiles of Biomarkers of Neuroinflammation and Neuronal Injury in the CSF of HAM/TSP Patients and HTLV-1 AC

**Figure 9:**
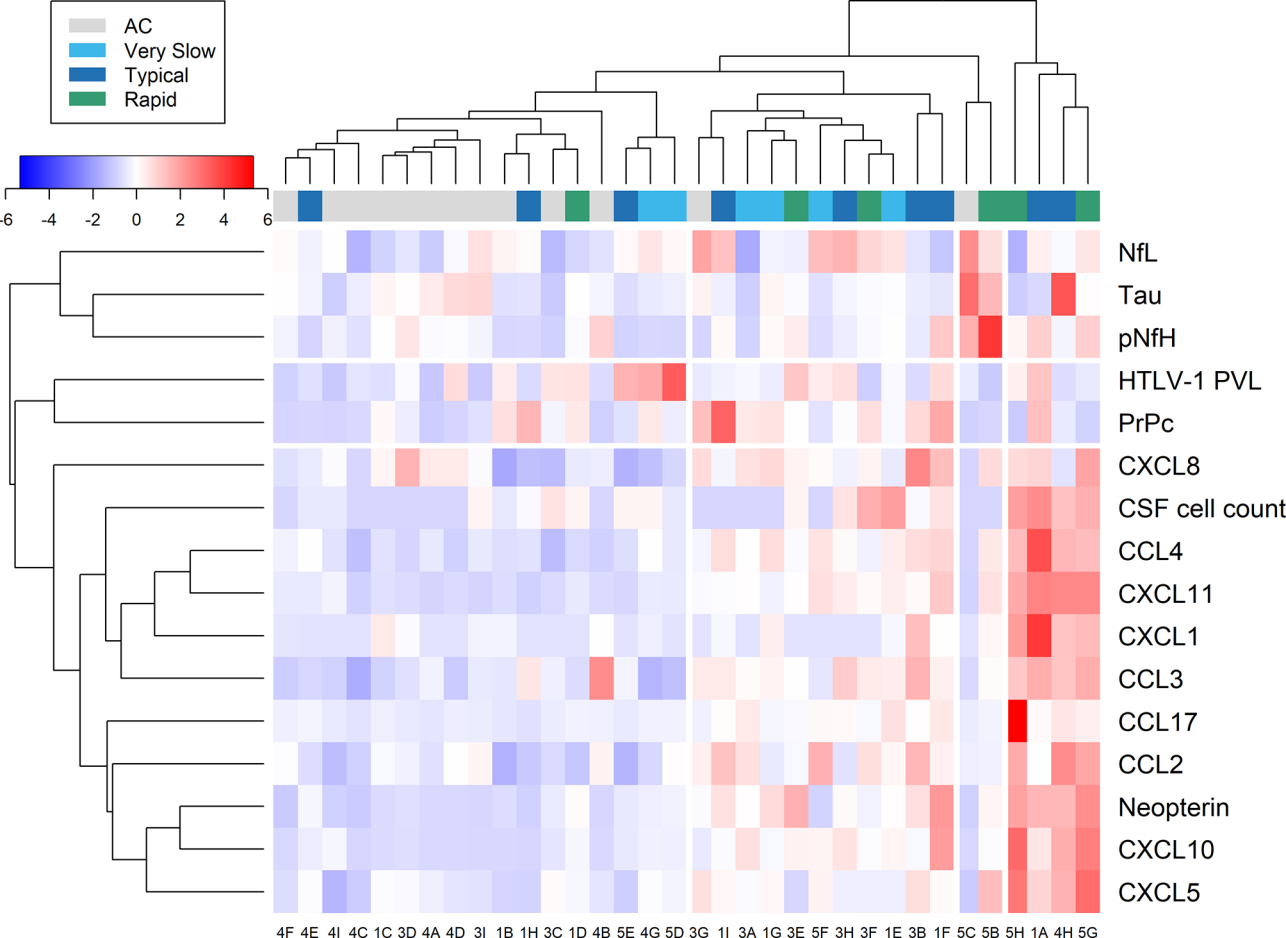
Relative expression of markers of neuroinflammation and neuronal injury. Data from cerebrospinal fluid (CSF) analysis of HTLV-1 asymptomatic carriers (AC) and HAM/TSP patients identified with very slow (light blue), typical (dark blue), and rapid (green) disease progression were evaluated by heatmap analysis. This included CSF cell counts, HTLV-1 proviral load (PVL), and the concentration of neurofilament light (NfL) and phosphorylated heavy (pNfH) chains, total Tau protein, cellular prion protein (PrPc), neopterin, and inflammatory chemokines (CCL2, CCL3, CCL4, CCL17, CXCL1, CXCL5, CXCL8, CXCL10, CXCL11).

Heatmap analysis of CSF data, including biomarkers of neuronal injury, chemokines, neopterin, CSF cell counts, in addition to HTLV-1 PVL in PBMC, according to the neurological status of patients and the speed of HAM/TSP progression resulted in distinct profiles (**Figure 9**). Biomarkers of neuroinflammation, neurodegeneration, and HTLV-1 PVL clustered separately, suggesting distinct dynamics of these parameters.

Most HTLV-1 AC (11 of 13) clustered closely. This group was characterized by low relative expression of inflammatory chemokines and neopterin and normal CSF cell counts. On the other hand, no specific pattern was observed for biomarkers of neuronal death (NfL, pNfH, PrPc, and Tau protein) and HTLV-1 PVL. Two HTLV-1 AC that clustered separately (**Figure 9**, patients 5C and 3G) had normal CSF cell counts and total protein levels but presented with high HTLV-1 PVL in PBMC (both with approximately 4.5%) and elevated neopterin levels in CSF and serum. However, the neopterin_CSF/Serum_ ratio was < 1.0. However, these individuals did not develop clinical signs of neurological alterations, including cognitive impairment, in three years following sample collection. Patient 5C showed increased tTau (733.1 pg/mL), NfL (2,329 pg/mL) and pNfH (677.3 pg/mL), and patient 3G displayed high NfL concentration (2,165 pg/mL), suggesting the presence of subclinical neuronal injury. Conversely, low relative expression of biomarkers of neuroinflammation was observed.

HAM/TSP patients that clustered close to HTLV-1 AC were characterized by normal CSF cell counts and total protein levels, moderate relative expression of biomarkers of inflammation and neurodegeneration, and mild neurological impairment (**Figure 9**).

The analysis according to the speed of HAM/TSP progression did not reveal any specific pattern for biomarkers of neuroinflammation and axonal degeneration. Only two patients had an intense elevation of tested biomarkers of neuronal death, an HTLV-1 AC (Patient 5C) and a 61-year-old woman (Patient 5B) with early HAM/TSP onset (14 months) and mild disability, which showed high levels of tTau (490.5 pg/mL), NfL (1,738 pg/mL), pNfH (1,222 pg/mL). Despite normal CSF cell counts, patient 5B had HTLV-1 PVL in CSF cells (9.37%) 15-fold higher than in PBMCs (0.60%). On the other hand, a cluster of patients with typical or rapid disease progression showed pleocytosis and intense relative expression of neopterin and inflammatory chemokines. Among them, patient 4H had 14 years of disease, the highest concentration of tTau (813.4 pg/mL), and increased neopterin levels, although NfL and pNfH levels were unaltered. Patient 5G had moderate neurological disability and was the only one to have a CSF/serum ratio ≥ 1.5 for CCL3 (ratio = 4.16) and had the highest ratio values for CXCL8 (ratio = 18.53) and CXCL10 (ratio = 19.38). CSF analysis also revealed mild elevation of total proteins (63.8 mg/dL), mononuclear pleocytosis (8 cells/mm^3^), and increased pNfH levels (554.3 pg/mL) confirming ongoing CNS inflammation.

## Discussion

HAM/TSP is a chronic demyelinating inflammatory disease that typically presents with a slow course. Despite the correlation between HAM/TSP disability severity and illness duration, a small proportion of individuals show a very slow progression with overall mild motor impairment. On the other hand, faster disease progression at early stages may represent an important indicator of deterioration in the clinical picture of HAM/TSP. Thus, evidence-based prognosis may improve the decision for therapeutic intervention and consecutive follow-up. Neopterin and CXCL10 have been proposed as biomarkers for HAM/TSP activity and disease prognosis since increasing CSF levels of these factors correlate with rising scores in the Osame Motor Disability Score (39–41). According to Sato et al. (40), HAM/TSP patients with rapid progression had CSF levels of neopterin and CXCL10 higher than 44 nmol/L and 4400 pg/mL, respectively. In turn, individuals displaying very slow progression were characterized by CSF neopterin and CXCL10 below 5.5 nmol/L and 320 pg/mL, respectively. Consequently, those within these upper and lower cut-offs would likely have the typical form of HAM/TSP. Unfortunately, such cut-offs for neopterin and CXCL10 levels in CSF have not agreed with our study group’s characterization of HAM/TSP progression as assessed by the IPEC-2 disability scale. However, the usefulness of neopterin and CXCL10 as biomarkers of inflammation in predicting the speed of HAM/TSP progression was confirmed. In this context, neopterin_CSF/Serum_ ratio differentiated most patients with very slow and typical/rapid HAM/TSP progression. Indeed, patients with a moderate and severe neurological disability had typical or rapid disease progression, while patients with very slow progression showed overall mild neurological impairment (data not shown). In addition, to overcome differences implicit to neopterin quantification by distinct methods, we propose using neopterin_CSF/Serum_ ratio instead of concentration cut-offs.

Neopterin concentration commonly correlates with cell-mediated immune processes during inflammation (47, 48). Neopterin was elevated in the CSF of HAM/TSP patients with early disease onset (from 5 months to 3 years) as well as after 20 years of disease. A proportion of HTLV-1 AC also displayed elevated neopterin in CSF and/or serum samples, although at levels similar to those observed in HTLV-1 seronegative individuals with non-inflammatory non-degenerative neurological disease (normal-pressure hydrocephalus). White matter brain lesions on magnetic resonance imaging have been shown in HTLV-1 AC (51), and this may explain the mild elevation of neopterin in these individuals. However, neopterin_CSF/Serum_ ratio values among HTLV-1 AC were < 1.0, indicating that inflammation was more pronounced or restricted to the periphery.

Distinct processes of leukocyte biology such as activation, adhesion, tissue homing, and migration are controlled by chemokines, which are small proteins with chemoattractant properties. Inflammatory chemokines are induced during the immune responses at sites of infection and recruit well-defined leukocyte subtypes (52, 53). In our cohort, elevated serum levels of CXCL9, CXCL10, and CXCL11 in HAM/TSP patients corroborated with previous studies (39, 54, 55). In physiological conditions, these chemokines are undetectable in most non-lymphoid tissues but are strongly induced upon IFN-γ signaling, infection, or tissue injury (53, 56). In HAM/TSP, these chemokines are involved in recruiting CXCR3^+^ cells to the CNS, which are mostly IFN-γ^+^CD4^+^ T-cells infected by HTLV-1 (17, 50). The IFN-γ secreted by these cells, in turn, stimulate the production of CXCL10 by astrocytes, the main source of this chemokine in the CNS, creating a positive feedback in chronic CNS inflammation associated with HAM/TSP (17, 57). We observed that a proportion of HTLV-1 AC also showed a relative elevation in CSF levels of CCL2, CXCL8, and CXCL10. However, slightly higher levels of these chemokines are constitutively present in the CSF compared to serum, as shown in the control group, which corroborates data in the literature from control groups of individuals without neurological conditions or CNS infection (58–60). On the other hand, such control groups do not account for the impact of chronic inflammatory responses that control HTLV-1 burden in the peripheral blood, which rise serum baseline levels of chemokines in infected individuals, as demonstrated by higher serum levels of CCL2 and CXCL8 in HTLV-1 infected individuals in general, and of CXCL10 in HAM/TSP patients. Consequently, this lowered the CSF to serum chemokine ratio since a proportional elevation was not observed in CSF counterparts. Nevertheless, elevated CCL2 and CXCL8 levels in the CSF of HTLV-1 AC are compatible with microglial/astrocytic activation (61, 62), which was likely induced by infected cells also present in the CSF of these individuals. CCL2 expression was shown in perivascular infiltrating cells and the vascular endothelium in spinal cord lesions of HAM/TSP patients (63). In addition, CXCL8 can enhance the microglial production of matrix metalloproteinases, thus leading to BBB disruption (64). Altogether, these events support the hypothesis that these chemokines may play a role in increasing the permeability of the BBB, therefore favoring HAM/TSP development.

CCL2 binds to CCR2 and directs monocyte/macrophage infiltration into the CNS (65), while CXCL10 recruits Th1 and CD8^+^ CTLs expressing CXCR3 to sites of inflammation. In turn, CXCL8 is a ligand of CXCR1 and CXCR2, which are expressed in neutrophils, monocytes, NK cells, CD4^+^ and CD8^+^ T-cells (53, 57). However, only in the group of HAM/TSP patients was observed a correlation between CSF levels of CXCL10 and pNfH, a biomarker for neuronal injury, and also with other markers of neuroinflammation, such as the neopterin_CSF/Serum_ ratio and CSF cell counts. In addition, HAM/TSP patients present higher HTLV-1 PVL in PBMC and an increased frequency of CXCR3^+^ cells in the peripheral blood in both infected (Tax^+^) and uninfected CD4^+^ T-cells. Therefore, HAM/TSP patients have a stronger potential of infected cells to migrate towards the CNS in response to the CXCL10 concentration gradient, confirming the role of this chemokine in the chronic CNS inflammation associated with HAM/TSP.

It has been shown that CCL2/CCR2 signaling is upregulated and plays a role in the pathogenesis of neurodegenerative disorders, such as Alzheimer’s disease, multiple sclerosis, HAND, and in the experimental autoimmune encephalomyelitis, a murine model for inflammatory demyelinating diseases (52, 65). CCL2 can be produced by several CNS cell types, particularly astrocytes, microglia, and endothelial cells (65). In patients with multiple sclerosis, the concentration of CXCL10 and CCL2 in CSF correlates with disease activity, returning to normal levels upon therapy (66, 67). However, the sole increased relative levels of inflammatory chemokines CXCL10 and CCL2 in the CSF of HTLV-1 AC did not result in pleocytosis or elevation of other biomarkers of neuroinflammation, as observed in non-infected individuals, suggesting that a combination of multiple events more likely triggers the development of HAM/TSP.

Free PrPc in the CSF, but not in the serum, represents a specific biomarker for neurocognitive impairment and CNS dysfunction ​​in individuals with HAND (38). The PrPc is constitutively expressed in neurons and localizes at the extracellular plasma membrane (36, 37). Therefore, in case of neuronal damage, PrPc is released into the surrounding environment, thus indicating CNS injury. This increase in soluble PrPc in patients with HAND has been associated with high CCL2 levels (38). However, although CCL2 was increased in the CSF of HAM/TSP patients, it did not correlate with PrPc levels (data not shown). The reason for increased CSF levels of PrPc is not clear but may be related to the death of neuronal cells or even the killing of HTLV-1-infected cells.

Inflammatory chemokines as CCL3, CCL4, and CXCL5 were also at significantly higher levels in the CSF of HAM/TSP patients. However, their relative levels were lower in comparison to the serum. CCL3 and CCL4 can bind to CCR5, which is mainly expressed by Th1 cells, CD8^+^ CTLs, and monocytes. In turn, CXCL5 is a ligand of CXCR2, which is expressed by neutrophils (53). CXCR2 is also a receptor for CXCL8, which was at higher levels in the CSF of HTLV-1 AC and HAM/TSP patients. However, polymorphonuclear cells were not detected in the CSF of these individuals, and infiltrating cells were predominantly mononuclear cells. Thus, it is possible that CSF levels of CXCL5 and CXCL8, particularly in HAM/TSP patients, were not physiologically relevant to induce neutrophil infiltration into the CNS. However, we were not able to determine this *in vitro*.

Except for PrPc, none of the biomarkers of neuronal injury differentiated HTLV-1 AC and HAM/TSP patients. The chronic and slow course of HAM/TSP was likely responsible for those findings. Another hypothesis is that none of the patients were suffering from an acute process of neuronal destruction. Tau protein is responsible for stabilizing axonal microtubules, and it is mainly expressed in unmyelinated cortical axons. In pathological conditions, Tau self-assembles into filamentous structures that end up forming neurofibrillary tangles, which cause axonal damage (31). Although elevated Tau levels in CSF correlate with an increased rate of neurodegeneration, our data corroborated with a previous observation that HAM/TSP patients have normal Tau levels (68), which is in agreement with the chronic and slowly progressive neurological damage in HAM/TSP.

Neurofilament proteins have been shown as promising biomarkers of neuronal injury of myelinated axons (30, 69). In multiple sclerosis, CSF levels of neurofilament components indicate disease activity, clinical progression and have been used to monitor disease-modifying therapies (29, 67). While increasing NfL levels predicted motor deterioration in the progressive forms of multiple sclerosis, pNfH levels correlated with disease activity. In relapsing-remitting multiple sclerosis, NfL levels reflected the acute axonal damage as they increase during relapses (28, 67).

Half of HTLV-1 AC and two-thirds of HAM/TSP patients had elevated NfL levels in CSF in the study population. This corroborates with the hypothesis of subclinical neuroinflammation in HTLV-1 AC. However, as observed for Tau protein, slow progression of HAM/TSP might have abrogated the usefulness of NfL and pNfH quantification to differentiate between HTLV-1 AC and HAM/TSP patients, as also shown by Alberti et al. (70). In amyotrophic lateral sclerosis, NfL levels are normal in the asymptomatic stage and increase at early symptoms onset (71). As most HAM/TSP patients had a long-term disease, it is possible that a significant increase of these factors at periods of intense inflammation was missed.

Amyotrophic lateral sclerosis is characterized by degeneration of the upper and lower motor neurons, and increased NfL levels have been associated with faster disease progression (27). NfL elevation in CSF was strongly associated with the dementia stage in Alzheimer (72), and in HIV carriers it preceded HAND development, which is characterized by cognitive and motor impairment (73). Moreover, increased NfL in the CSF indicated ongoing axonal injury in neurologically asymptomatic HIV carriers with low CD4 counts (30). Moreover, NfL levels correlated with neopterin and albumin ratio, suggesting that neuroinflammation is associated with BBB permeability and axonal injury in HAND development (23, 30). In our study, a strong correlation was observed between the neopterin_CSF/Serum_ ratio and increasing levels of pNfH in the CSF of HAM/TSP patients. Heavily phosphorylated NfH seems to have a longer half-life than NfL (74), which may benefit the identification of limited neuronal damage in HAM/TSP when associated with markers of neuroinflammation.

The study faced limitations such as the small study population, the absence of HAM/TSP patients with the subacute form of the disease, and missing data of HTLV-1 PVL in CSF. Notwithstanding, we confirmed the chronic CNS inflammation in HAM/TSP patients indicated by the higher concentration of neopterin and CXCL10 in the CSF. Interestingly, this revealed subclinical inflammation in the CNS of a small proportion of HTLV-1 AC, which may need closer follow-up. Conversely, biomarkers of neuronal injury (Total Tau protein, NfL and pNfH, and PrPc) were not helpful to define HAM/TSP prognosis. Still, they evidenced neuronal death associated with exacerbated inflammation, particularly indicated by neopterin levels. Therefore, quantification of neopterin in the CSF and serum to calculate the neopterin_CSF/Serum_ ratio, in addition to the quantification of pNfH, may contribute to define HAM/TSP prognosis into very slow and typical/fast disease progression and to identify HTLV-1 AC in risk of progression to HAM/TSP. However, studies in other cohorts from distinct endemic regions and with a larger number of individuals need to be carried out in order to confirm these findings.

## Data Availability Statement

The raw data supporting the conclusions of this article will be made available by the authors, without undue reservation.

## Ethics Statement

The studies involving human participants were reviewed and approved by the committee of ethics in research of the Instituto Nacional de Infectologia Evandro Chagas. The patients/participants provided their written informed consent to participate in this study.

## Author Contributions

OME designed the study. ACCBL, MASDL, MTTS, and AQCA conducted the neurological assessment and clinical follow-up of patients. FSS, NLF, RCT, YCPG, and ILS-F performed assays and data analysis. OME and YCPG carried out statistical analysis. OME, FSS, YCPG, JE-L, ACCBL, MASDL, MTTS, and AQCA drafted and corrected the manuscript.

## Funding

OME was awarded with grants in the INOVA Program - Fundação Oswaldo Cruz (Grant number VPPCB-008-FIO-18-2-39). NLF and YCPG were granted with Master’s scholarships from the Fundação Oswaldo Cruz and the Coordination for the Improvement of Higher Education Personnel (CAPES) of the Brazilian Ministry of Education, respectively. This study was partially supported by the Coordination for the Improvement of Higher Education Personnel (Coordenação de Aperfeiçoamento de Pessoal de Nível Superior - CAPES) - Finance Code 001.

## Conflict of Interest

The authors declare that the research was conducted in the absence of any commercial or financial relationships that could be construed as a potential conflict of interest.

## Publisher’s Note

All claims expressed in this article are solely those of the authors and do not necessarily represent those of their affiliated organizations, or those of the publisher, the editors and the reviewers. Any product that may be evaluated in this article, or claim that may be made by its manufacturer, is not guaranteed or endorsed by the publisher.
