# Supplementary material for: Following the Clues: Usefulness of Biomarkers of Neuroinflammation and Neurodegeneration in the Investigation of HTLV-1-Associated Myelopathy Progression
Source: Front Immunol. 2021 Oct 26;12:737941. doi: 10.3389/fimmu.2021.737941 (PMC8576432; doi:10.3389/fimmu.2021.737941)
Supplement: Supplementary file 1 [file DataSheet_1.pdf]

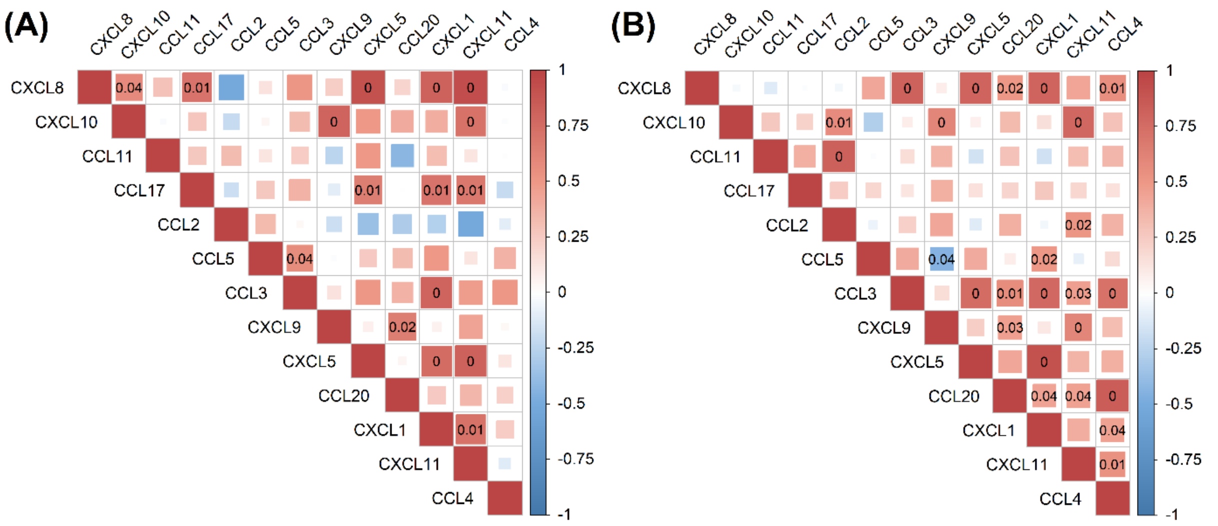

Supplementary Figure 1. Correlation analysis between the concentration of chemokines in serum samples. Chemokines were quantified with a multiplex cytometry bead-based immunoassay in serum samples from **(A)** HTLV-1 asymptomatic carriers (n=13) and **(B)** HAM/TSP patients (n=21). Correlation analysis was carried out with Spearman's correlation rank with data from chemokines that presented detectable levels in at least half of the samples. Correlation coefficient in bivariate analyses is represented by the color intensity of squares, which are shown in red for positive correlation and in blue for an inverse correlation. The size of the squares corresponds to the level of statistical significance of the correlation (shown inside the squares), and associations with *p*-values < 0.05 were considered significant.

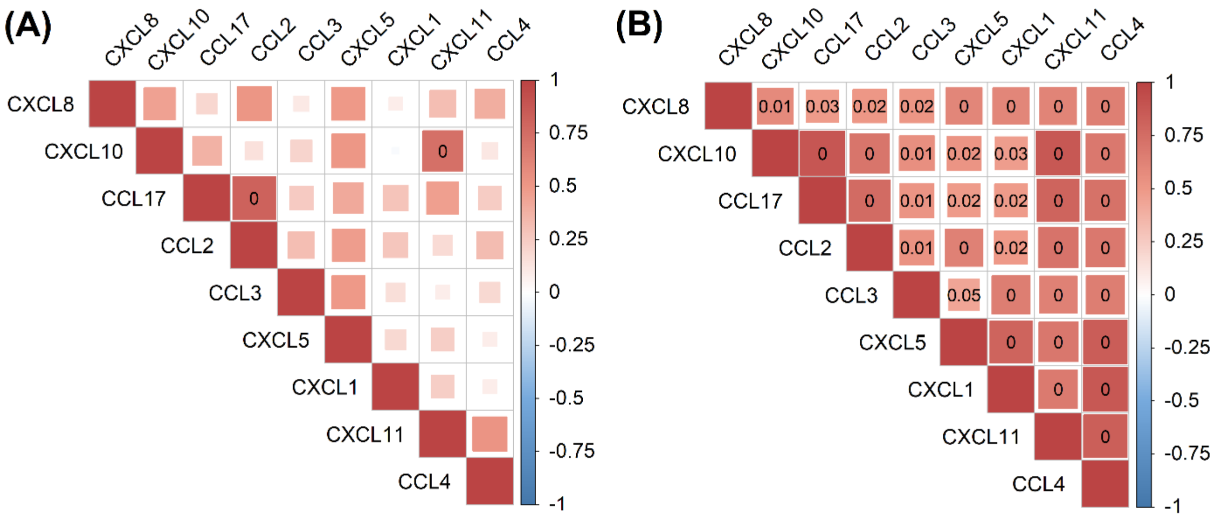

Supplementary Figure 2. Correlation analysis between the concentration of chemokines in the cerebrospinal fluid. Chemokines were quantified with a multiplex cytometry bead-based immunoassay in serum samples from **(A)** HTLV-1 asymptomatic carriers (n=13) and **(B)** HAM/TSP patients (n=21). Correlation analysis was carried out with Spearman's correlation rank with data from chemokines that presented detectable levels in at least half of the samples. Correlation coefficient in bivariate analyses is represented by the color intensity of squares, which are shown in red for positive correlation and in blue for an inverse correlation. The size of the squares corresponds to the level of statistical significance of the correlation (shown inside the squares), and associations with *p*-values < 0.05 were considered significant.

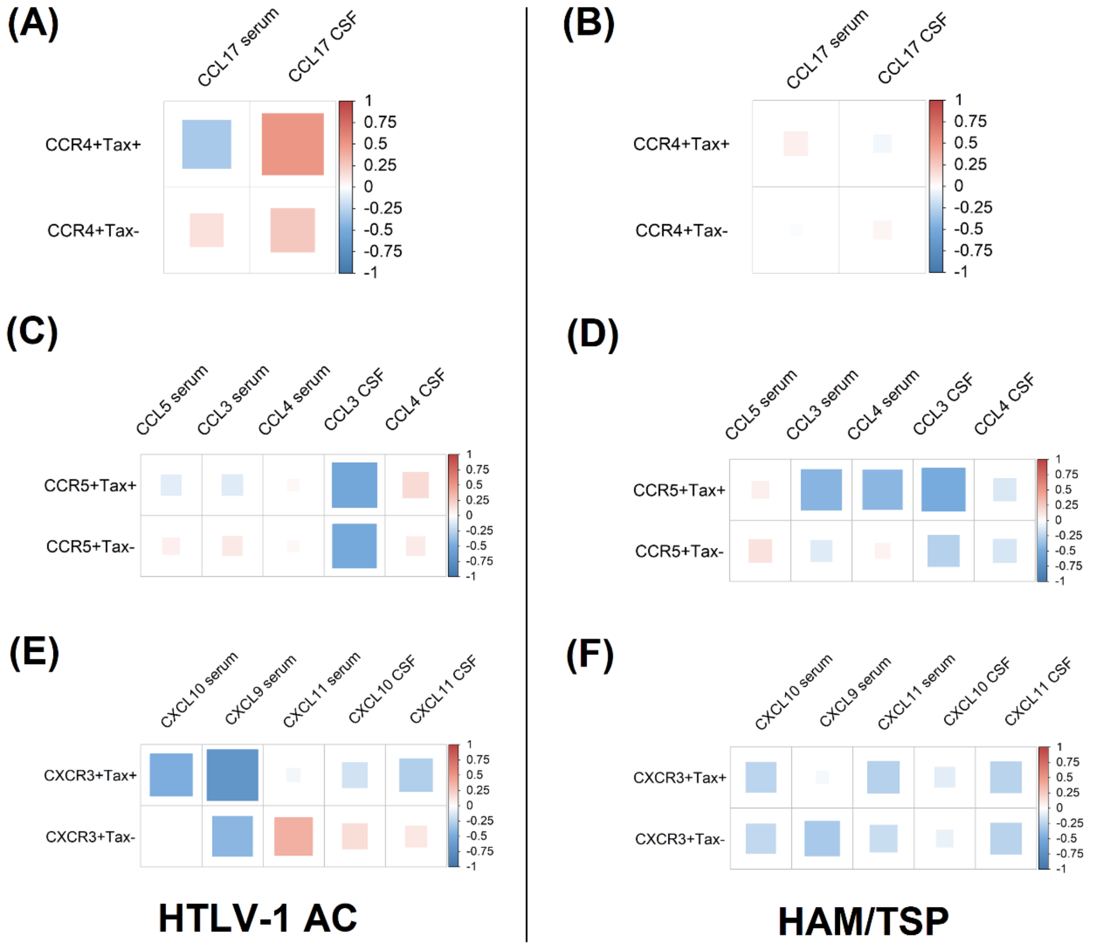

Supplementary Figure 3. Correlation analysis between the concentration of chemokines in the serum and cerebrospinal fluid and the frequency of expression of chemokine receptors in $CD4^{+}$ T-cells. Chemokines were quantified with a multiplex cytometry bead-based immunoassay in serum samples from HTLV-1 asymptomatic carriers (AC) and HAM/TSP patients. Infected (Tax^{+}) and uninfected (Tax^{-}) $CD4^{+}$ T-cells from the peripheral blood of HTLV-1 AC (n=9) and HAM/TSP patients (n=15) were identified by flow cytometry analysis Frequencies of cells expressing the chemokine receptors **(A,B)** CCR4, **(C,D)** CCR5, and **(E,F)** CXCR3 were determined within the populations of Tax^{+} and Tax^{-} $CD4^{+}$ T-cells. Correlation analysis was carried out with Spearman's correlation rank with data from chemokines that presented detectable levels in at least half of the samples. Correlation coefficient in bivariate analyses is represented by the color intensity of squares, which are shown in red for positive correlation and in blue for an inverse correlation. The size of the squares corresponds to the level of statistical significance of the correlation (shown inside the squares), and associations with *p*-values < 0.05 were considered significant.
